# Supplementary material for: Associations between area-level socioeconomic disadvantage and COVID-19 disease consequences in Sydney, Australia: A retrospective cohort analysis
Source: PLoS One. 2026 Apr 3;21(4):e0334206. doi: 10.1371/journal.pone.0334206 (PMC13048482; doi:10.1371/journal.pone.0334206)
Supplement: Attachment — Submitted filename: Response to Reviewers.pdf [file pone.0334206.s003.pdf]

17 February 2026

Eric HY Lau, Ph.D.
Academic Editor
PLOS One

Dear Associate Professor Lau

Re: Research article submission – "Associations between area-level socioeconomic disadvantage and COVID-19 disease consequences in Sydney, Australia: A retrospective cohort analysis"

In the table below, we respond to each point raised by the academic editor and reviewer.

Section/lines in original manuscript	***Recommendations***	***Response***
Methods<br><br>(lines 112-114)	The methods section should specify how the IRSD is calculated and the geographic scale (e.g., SA1, SA2) that this is applied at.	We have added a sentence and reference explaining how IRSD scores are calculated: "An IRSD score for a postal area is a weighted composite measure of 16 economic and social disadvantage indicators for that postal area, including the percentage of people aged 15 years and over who have no educational attainment, and the percentage of people who are unemployed [16]."
Methods<br><br>(lines 95-100)	It is unclear whether self-reported rapid antigen tests (RATs) were included, which could affect case ascertainment.	We have clarified in the Methods section that self-reported RAT results were included:<br><br>"From 12 January 2022, self-reporting of positive COVID-19 rapid antigen tests (RATs) was mandatory and could be done via the 'Service NSW' app or website, or by phone."<br><br>We have also provided the proportion of self-reported positive cases in the Results section and addressed variation in case ascertainment in the Discussion.
Results<br><br>Figures 1-4	The current colour scale is difficult to interpret and should be revised. Suggest using multiple colours (for example - red, orange, green).	Red, orange and green are difficult to distinguish for many people with colour vision deficiency. A monochromatic colour scheme is recommended by accessible publishing guidelines, such as WCAG. A monochromatic colour scheme works well when printed in greyscale.

UNSW SYDNEY NSW 2052 AUSTRALIA
T +61 2 9385 1000 | F +61 2 9385 0000 | ABN 57 195 873 179 | CRICOS Provider Code 00098G

Section/lines in original manuscript	*Recommendations*	*Response*
Results	This is acknowledged a limitation in the discussion – However, stratifying results by year (2020/2021/2022) could reveal shifts in associations.	Thank you for this suggestion. We have addressed temporal heterogeneity by extending the analysis to include adjustment for pandemic phase, rather than calendar year, to capture major shifts in dominant SARS-CoV-2 variants, population immunity, and other factors.<br><br>Specifically, we classified cases into three pandemic phases based on diagnosis date: Ancestral (March 2020 to June 2021), Delta (June to November 2021), and Omicron (December 2021 to February 2022). Pandemic phase was included as a categorical covariate in the logistic regression models, which substantially improved overall model fit for both hospitalisation and death.<br><br>To assess whether socioeconomic gradients differed over time, we fitted pooled models including interaction terms between area-level socioeconomic disadvantage (IRSD) and pandemic phase. These interaction terms were not statistically significant for either hospitalisation or death, indicating no evidence that the association between socioeconomic disadvantage and disease severity varied across the three pandemic phases. Accordingly, final models are presented without the interaction terms.<br><br>The Abstract, Methods, Results, and Discussion sections have been updated to reflect these additional analyses and implications.
Methods - Figure 1	Zoomed in call out box of city and inner Sydney suburbs would provide better detail on heterogeneity of IRSD	Adding additional inset maps for the inner city and other densely populated areas with small postal areas would make the map very cluttered. However, we agree it would be helpful to provide more detail on the heterogeneity of the IRSD variable. We have therefore provided a measure of the heterogeneity in the text:<br><br>"IRSD scores are standardised to have a mean of 1,000 and a standard deviation of 100 across Australia. In the study area, IRSD scores ranged from 711 to 1,122, with a mean of 1,029 and a standard deviation of 78."

Section/lines in original manuscript	***Recommendations***	***Response***
Results	An epidemic curve would contextualize the temporal dynamics of cases	We have added an epidemic curve with pandemic phases (Ancestral, Delta, Omicron) indicated to contextualise the temporal dynamics of COVID-19 cases over the study period (Fig 2).
Introduction<br><br>(lines 51-61)	These two paragraphs can be difficult to follow for those unfamiliar with the pathways described by Katikireddi, et al. 2021 - Suggest rewording to clarify and improve flow of text.	We have reworded this section of the Introduction to describe the Katikireddi et al. framework using more accessible language.
Methods<br><br>(lines 93-94)	Suggest specifying what percent of the population (including eligible age groups) to add clarity.	We have revised the Methods section to specify the percent of the eligible population aged ≥16 years that had received two vaccination doses.
Methods<br><br>(line 97)	Suggest including mention of the private health system in Australia.	We have clarified that PCR testing was provided by both public and private providers, and that some hospitalisations for COVID-19 were managed in private hospitals.
Methods<br><br>(lines 101-103)	It is unclear why this sentence is included in the methods.	We have moved discussion of infection-control measures in aged care settings to the Discussion, as it is of relevance to the observed number of COVID-19 hospitalisations and fatalities in older adults.
Results	How many cases did not include a valid postal area and/or were acquired overseas.	We have clarified that 2,527 cases were excluded because they were acquired overseas. All cases had a valid postal area after linking them to IRSD scores.
Results<br><br>Figures 2-4	Suggest rounding decimal places of legend to improve readability.	We have reduced the number of decimal places to one.
Discussion<br><br>(lines 223-226)	Suggest including reference to support these claims.	In our original submission, the references were provided in the subsequent paragraph. We have merged the two paragraphs to make it clearer that those references are associated with these claims.
Discussion	Suggest adding a brief overview of vaccination uptake to provide context.	We have added the date COVID-19 vaccination became available, as well as coverage at the ends of the Delta and Omicron pandemic phases, to the Methods section.
Supplementary information	Suggest including overview of demographics of cases included in analysis compared to local population.	We have provided demographics of cases included in the analysis compared to the local population in Table 1.

Commented [ET1]: agree this is clearer

Commented [ET2R1]: And private health system point is well covered too

Section/lines in original manuscript	***Recommendations***	***Response***
Table 2	What is the range for the IRSD score? The logistic regression estimated the effect of 1 unit IRSD increase but that could be a very small difference. Please consider rescaling the IRSD score in the model to improve interpretation.	Thank you for the suggestion. We have rescaled the IRSD variable to 100-point intervals and updated the Methods section to provide more background.
Table 2	The authors may consider using 20-49 years as reference group, as COVID-19 deaths were rare among 0-19 years which may lead to unstable estimates.	We have changed the reference category for the age variable in both logistic regression models to be 20–49 years.

Sincerely,

Christopher Standen (corresponding author, on behalf of all co-authors)
Research Fellow
International Centre for Future Health Systems
University of New South Wales
Email: c.standen@unsw.edu.au | Phone: +61 2 9065 6081
