# Supplementary material for: Associations between area-level socioeconomic disadvantage and COVID-19 disease consequences in Sydney, Australia: A retrospective cohort analysis
Source: PLoS One. 2026 Apr 3;21(4):e0334206. doi: 10.1371/journal.pone.0334206 (PMC13048482; doi:10.1371/journal.pone.0334206)
Supplement: Attachment — Submitted filename: Review report PONE-D-25-51852.docx [file pone.0334206.s001.docx]

**Manuscript: PONE-D-25-51852**

**Reviewer name:** Selina Ward (<https://orcid.org/0000-0002-7776-8419> )

**Review date:** 10/12/2025

**Summary:**

Thank you for this submission - This paper investigates the relationship between area‑level socioeconomic disadvantage and COVID‑19 outcomes in Greater Sydney, Australia, focusing specifically on disease severity once infected. Using a retrospective cohort design, the authors analysed over 780,000 confirmed and probable cases between March 2020 and February 2022. They measured disadvantage via the Index of Relative Socio‑Economic Disadvantage (IRSD) and applied logistic regression models adjusted for age and gender. The findings show that lower IRSD scores (greater disadvantage) were consistently associated with higher odds of hospitalization and death, even in a setting with universal public hospital care. The authors conclude that inequities in comorbidity burden and possibly differences in care access underlie these disparities, and they recommend prioritizing chronic disease prevention and management in disadvantaged communities.

There has been similar work (Guajardo, et al. 2025) conducted examining how socioeconomic and demographic factors influenced COVID-19 outcomes (testing, infection, hospitalisation and deaths) in the Sydney Local Health District (SLHD). Key difference between these two works is the focus on the impact of public health restrictions (strict vs relaxed public health restrictions) on these outcomes with regards to socioeconomic and demographic factors and that this study focuses on a greater geographical area (beyond Sydney city) and larger time-period.

**Placed in the context of existing literature, this study** **reinforces global evidence that socioeconomic disadvantage amplifies vulnerability to severe COVID‑19 outcomes**. While similar associations have been documented nationally (Flavel, et al. 2022) and in Australian states (Queensland – Ward, et al. 2023; Victoria – Roder, et al. 2022), the originality of this work lies in its large-scale, population-level analysis, highlighting that inequities persist even when financial barriers to care are minimized (in the context of universal free health care in Australia). **Thus, the paper is more confirmatory than novel, but it provides important local evidence that strengthens the broader narrative of social determinants of health in pandemic outcomes.**

**Strengths of the work:**

- Large sample size and time-period: Over 780,000 cases between 2020 and 2022.
- Clear public health relevance: Findings emphasize the need for targeted interventions in disadvantaged communities.
- Policy significance: Demonstrates inequities even in a universal healthcare setting, underscoring systemic issues beyond access.

**Major and minor issues and considerations:**

| Section/lines | Issue/weakness | Severity of impact | Recommendations |
| --- | --- | --- | --- |
| Methods  (lines 112-114) | Insufficient detail on IRSD | Major | The methods section should specify how the IRSD is calculated and the geographic scale (e.g., SA1, SA2) that this is applied at. |
| Methods  (lines 95-100) | Case definition clarity | Major | It is unclear whether self-reported rapid antigen tests (RATs) were included, which could affect case ascertainment. |
| Results  Figures 1-4 | Colour scale for maps | Major | The current colour scale is difficult to interpret and should be revised. Suggest using multiple colours (for example - red, orange, green). |
| Results | Temporal sub-analysis | Major | This is acknowledged a limitation in the discussion – However, stratifying results by year (2020/2021/2022) could reveal shifts in associations. |
| Methods - Figure 1 | Zoomed in call out box of city and inner Sydney suburbs | Minor | Zoomed in call out box of city and inner Sydney suburbs would provide better detail on heterogeneity of IRSD |
| Results | Epidemic curve of cases over time | Minor | An epidemic curve would contextualize the temporal dynamics of cases |
| Introduction  (lines 51-61) | Flow of text | Minor | These two paragraphs can be difficult to follow for those unfamiliar with the pathways described by Katikireddi, et al. 2021 - Suggest rewording to clarify and improve flow of text. |
| Methods  (lines 93-94) | Clarification of vaccination rate | Minor | Suggest specifying what percent of the population (including eligible age groups) to add clarity. |
| Methods  (line 97) | Clarification of health care system | Minor | Suggest including mention of the private health system in Australia. |
| Methods  (lines 101-103) | Further elaboration | Minor | It is unclear why this sentence is included in the methods. |
| Results | Excluded data | Minor | How many cases did not include a valid postal area and/or were acquired overseas. |
| Results  Figures 2-4 | Legend of figure | Minor | Suggest rounding decimal places of legend to improve readability. |
| Discussion  (lines 223-226) | References | Minor | Suggest including reference to support these claims. |
| Discussion | Include brief overview of vaccination uptake in Sydney | Minor | Suggest adding a brief overview of vaccination uptake to provide context. |
| Supplementary information | Demographic information | Minor | Suggest including overview of demographics of cases included in analysis compared to local population. |

**References:**

Flavel, J. and Baum, F., 2022. The influence of socio‐economic conditions on the epidemiology of COVID‐19 in Australia. *The Medical Journal of Australia*, *216*(7), p.344.

Guajardo, M.G.U., Moore, C., Giannopoulos, V., Liu, H., Tickle, A., Adily, P., Mukumbang, F.C., Quinn, E., Cunich, M., Katz, I. and Page, A., 2025. The impact of contextual socioeconomic and demographic characteristics of residents on COVID-19 outcomes during public health restrictions in Sydney, Australia. Australian and New Zealand Journal of Public Health, 49(2), p.100228.

Roder, C., Maggs, C., McNamara, B.J., O'Brien, D., Wade, A.J., Bennett, C., Pasco, J.A. and Athan, E., 2022. Area‐level social and economic factors and the local incidence of SARS‐CoV‐2 infections in Victoria during 2020. Medical Journal of Australia, 216(7), pp.349-356.

Ward, S., Restrepo, A.C. and McHugh, L., 2023. Area-level geographic and socioeconomic factors and the local incidence of SARS-CoV-2 infections in Queensland between 2020 and 2022. *Australian and New Zealand Journal of Public Health*, *47*(6), p.100094.
